# Prevalence of thyroid disorders in a tertiary care hospital in Al Batinah North Governorate, Oman

**DOI:** 10.1016/j.dialog.2025.100246

**Published:** 2025-10-02

**Authors:** Vijaya Marakala, Gulam Saidunnisa Begum, Salima Al Maqbali, Elham Said Ahmed Al Risi

**Affiliations:** a Department of Biochemistry, National University of Science and Technology, College of Medicine and Health Sciences, Sohar campus, Oman; b Consultant Chemical Pathologist and metabolic diseases & Head, Department of Pathology and Blood bank Sohar hospital, Ministry of Health Sohar, Oman; c Chemical Pathologist Sohar Hospital, Ministry of Health Soha Sohar, Oman

**Keywords:** Thyroid dysfunction, Hypothyroidism, Hyperthyroidism, Lipids, Oman, Epidemiology

## Abstract

**Objectives:**

To assess the prevalence and demographic distribution of thyroid dysfunction in the Al Batinah North Governorate, Oman, and to examine associations between thyroid hormone levels and lipid profiles.

**Methods:**

This retrospective observational study was conducted at Sohar Hospital, a major referral centre in Al Batinah North. Electronic medical records of 40,390 patients who underwent thyroid function testing between 2020 and 2024 were reviewed. The prevalence of subclinical and overt hypothyroidism and hyperthyroidism, demographic distribution (age, gender), and associations between thyroid hormone levels and lipid profiles were analyzed. Only the first thyroid panel per patient was included to avoid duplication.

**Results:**

Of the 40,390 patients, 78.7 % were euthyroid, 13.9 % had subclinical hypothyroidism, 3.4 % had subclinical hyperthyroidism, 2.1 % had overt hyperthyroidism, and 2.0 % had overt hypothyroidism. Thyroid dysfunction was more prevalent among females (67.5 %) than males (32.5 %). Hypothyroidism was most frequent in individuals aged 0–17 years, while hyperthyroidism was more common in adults aged 36–50 years. A statistically significant inverse correlation was observed between free thyroxine (FT4) and serum lipid levels (cholesterol: *r* = −0.12, *p* < 0.001; triglycerides: *r* = −0.10, p < 0.001).

**Conclusions:**

Thyroid dysfunction, particularly subclinical hypothyroidism, was common in this hospital-based Omani cohort and disproportionately affected females. Associations with lipid abnormalities were statistically significant but weak. Findings should be interpreted with caution due to the retrospective design, hospital-based sampling, and incomplete adjustment for confounders. Population-based studies are required to establish true prevalence and long-term metabolic outcomes.

## Highlights

• Subclinical hypothyroidism was the most common thyroid dysfunction.
• Thyroid dysfunction was more frequent among females than males.
• Adolescents showed higher hypothyroidism; adults 36–50 years showed more hyperthyroidism.
• Weak but significant inverse correlations were found between FT4 and lipids.
• Hospital-based design limits generalizability; population studies are needed.

## 1 Introduction

Thyroid dysfunction is among the most common endocrine conditions worldwide and is associated with metabolic, cardiovascular, reproductive, and neuropsychological morbidity. It arises from disruptions in the hypothalamic–pituitary–thyroid axis, resulting in hypo- or hyperfunctioning states with systemic consequences [1,12,16]. Both conditions are strongly linked to lipid abnormalities and cardiovascular risk, making thyroid health an important public health concern.

Globally, the burden of thyroid disease is increasing, driven by aging populations, improved access to biochemical testing, and environmental factors including iodine intake. Hypothyroidism, particularly in its subclinical form, accounts for most cases, whereas hyperthyroidism is less common but clinically significant. Prevalence varies across regions: community studies in Europe and North America report hypothyroidism rates of 4–10 %, while Asian surveys generally suggest lower but heterogeneous rates. Such variation reflects differences in iodine nutrition, autoimmune predisposition, and healthcare practices.

In the Middle East, thyroid dysfunction is increasingly recognized as a regional health concern. Contributing factors include genetic susceptibility, a high burden of autoimmune disease, and historical iodine deficiency. Although Gulf countries implemented universal salt iodization in the 1990s, reports suggest persistent variability in iodine sufficiency, especially in rural and inland areas [3]. Hospital-based studies from Saudi Arabia, Kuwait, and the United Arab Emirates consistently identify subclinical hypothyroidism as the most common abnormality, particularly among women and individuals with comorbidities such as diabetes mellitus and chronic kidney disease. However, large-scale, population-based studies remain limited.

In Oman, thyroid dysfunction is frequently observed in clinical practice, but national prevalence estimates are lacking. Ministry of Health reports describe increasing referrals for endocrine disorders, reflecting both greater awareness and diagnostic capacity. Published studies remain fragmented, often restricted to subgroups such as pregnant women, patients with chronic kidney disease, or those with diabetes [4]. For example, research from Muscat demonstrated high rates of thyroid autoimmunity among women of reproductive age, while a study from Nizwa reported thyroid dysfunction among renal patients. While informative, these findings cannot be generalized to the wider Omani population.

The implications of thyroid dysfunction extend beyond metabolic disease. Hypothyroidism has been linked to infertility, miscarriage, and adverse pregnancy outcomes, while hyperthyroidism may increase risks of preterm delivery and low birth weight [14,15,18]. Thyroid dysfunction is also associated with psychiatric disorders and is more common among individuals with autoimmune conditions such as type 1 diabetes and Down syndrome [10,11,19]. Importantly, many cases are subclinical, detectable only through biochemical testing, yet even mild thyroid hormone disturbances can affect lipid metabolism and long-term cardiovascular health.

To address existing gaps, this study was conducted at Sohar Hospital, the largest referral centre in Al Batinah North Governorate, serving both urban and rural populations. The objectives were: (i) to determine the prevalence and demographic distribution of thyroid dysfunction including subclinical and overt hypothyroidism and hyperthyroidism—among patients undergoing thyroid function testing; and (ii) to evaluate associations between thyroid hormones (TSH, free T3, free T4), thyroid autoantibodies, and lipid profiles to explore potential metabolic implications.

It is important to note that this was a hospital-based, retrospective study. The findings therefore reflect referral and testing practices in a tertiary care setting and may not represent true population-level prevalence. Nonetheless, hospital-based data remain valuable for identifying patterns among patients most likely to present for evaluation, highlighting high-risk groups, and generating hypotheses for future community-based research. By providing region-specific evidence, this study seeks to advance understanding of thyroid dysfunction in Oman and similar Gulf settings while informing clinical practice and health policy.

## 2 Methods

### 2.1 Study design and setting

This retrospective, observational study was conducted at Sohar Hospital, a tertiary care facility operating under the Ministry of Health, Oman. The investigation was based on a review of electronic medical records of patients who underwent thyroid function testing between 2020 and 2024.

### 2.2 Study population and data sources

Patients were identified using the ALShifa+ Hospital Information System, and relevant data were extracted from the Department of Pathology records. The dataset included demographic information such as age and gender, results of thyroid function tests including serum TSH, FT3, and FT4, thyroid antibody status, lipid profile parameters, and relevant clinical history. Data extraction was carried out using a pre-designed form to ensure standardisation and completeness. All patient information was anonymised to maintain confidentiality in accordance with ethical research practices. To avoid duplication, only the first complete thyroid panel per patient during the study period was included in the analysis.

### 2.3 Inclusion and exclusion criteria

The study included patients who had complete thyroid function test results available—specifically TSH, FT3, and FT4 and who underwent testing within the defined study period. Patients were excluded if they had a prior diagnosis of thyroid disorders and were under active follow-up, were receiving thyroid-related medications, or had undergone thyroidectomy or radioiodine therapy before the testing period.

### 2.4 Biochemical assessments

All participants underwent a standardized panel of thyroid and metabolic tests. These included serum thyroid-stimulating hormone (TSH), free triiodothyronine (FT3), and free thyroxine (FT4). Where available, total T3 and total T4 values were also recorded. For patients suspected of autoimmune thyroid disorders, thyroid autoantibodies, particularly anti-thyroid peroxidase antibodies, were assessed in accordance with clinical guidelines [5]. All biochemical assays were performed using validated hospital laboratory methods and aligned with internationally accepted reference ranges. In addition, serum lipid profiles including total cholesterol, LDL cholesterol, HDL cholesterol, and triglycerides were measured to explore potential metabolic associations with thyroid dysfunction. All biochemical assays were performed in the Sohar Hospital laboratory using standardized, validated methods consistent with international guidelines. Although quality control procedures were in place, we were unable to formally assess potential assay drift or methodological changes across the study period, which remains a limitation.

### 2.5 Diagnostic classification criteria

Based on the serum levels of TSH, FT3, and FT4, patients were classified into five categories: euthyroid (normal values for all three parameters), subclinical hypothyroidism (elevated TSH above 5.6 μIU/mL with normal FT3 and FT4), overt hypothyroidism (elevated TSH above 5.6 μIU/mL accompanied by low FT3 and/or FT4), subclinical hyperthyroidism (suppressed TSH below 0.34 μIU/mL with normal FT3 and FT4), and overt hyperthyroidism (suppressed TSH below 0.34 μIU/mL with elevated FT3 and/or FT4).

### 2.6 Sample size

This retrospective study included all eligible patients who underwent thyroid function testing at Sohar Hospital between 2020 and 2024. A total of, 55,267 samples were gathered. 14,877 of them were excluded since they didn't meet the established diagnostic standards. With 40,390 records meeting the inclusion criteria, the final sample provided an accurate representation of thyroid function patterns within this population.

### 2.7 Ethical considerations

The study protocol received ethical approval from the Institutional Ethics Committee of College of Medicine and Health Sciences, National University of Science and Technology, Sohar, Oman (approval number: NU/COMHS/EBC0015/2024) and the Research and Ethical Review and Approval Committee, Directorate General of Health Services, North Batinah Governorate, Ministry of Health, Sultanate of Oman (approval number: MH/DGHS/NBG: MoH/CSR/24/29168). Patient data were anonymised prior to analysis, and no identifiable information was accessed or reported.

### 2.8 Statistical analysis

Data analysis was conducted using SPSS version 26 (IBM Corp., Armonk, NY, USA). Descriptive statistics (means, standard deviations, medians, and proportions) were used to summarize demographic, clinical, and biochemical variables. Prior to the analyses, missing values were managed using listwise deletion for thyroid function parameters to avoid misclassification. For lipid profiles and thyroid antibody testing, analyses were conducted on available subsets, and the number of patients included in each analysis has been reported. Prevalence estimates of thyroid dysfunction were calculated with 95 % confidence intervals. Associations between thyroid dysfunction categories and lipid parameters were assessed using independent *t*-tests, one-way ANOVA, chi-square tests, and correlation analyses (Pearson or Spearman, depending on data distribution). Age- and gender-stratified subgroup analyses were also performed to provide additional context. Due to limitations in the dataset, information on comorbidities was incomplete, and multivariable regression modeling could not be performed. Therefore, associations reported are unadjusted and should be interpreted cautiously. Effect sizes and 95 % confidence intervals were reported to aid interpretation. A two-tailed *p*-value <0.05 was considered statistically significant.

## 3 Results

### 3.1 Study population

A total of 40,390 patients who underwent thyroid function testing between 2020 and 2024 were included in the initial screening. Of these, 67.5 % (*n* = 27,267) were female and 32.5 % (*n* = 13,123) were male. The majority of participants were Omani nationals (95 %), reflecting the study's target population (Supplementary Table 1). The most represented age group was 36–50 years (30.5 %), while individuals aged 51–65 years constituted the smallest group (15 %) (Table 1).

**Table 1:** Demographic and thyroid function characteristics of patients (*n* = 40,390).

Variable	Category	n	%
Gender	Female	27,267	67.5
	Male	13,123	32.5
Age group (years)	0–17	6694	16.6
	18–35	9206	22.8
	36–50	12,322	30.5
	51–65	6076	15.0
	>65	6092	15.1

### 3.2 Thyroid function status

**Fig. 1:**
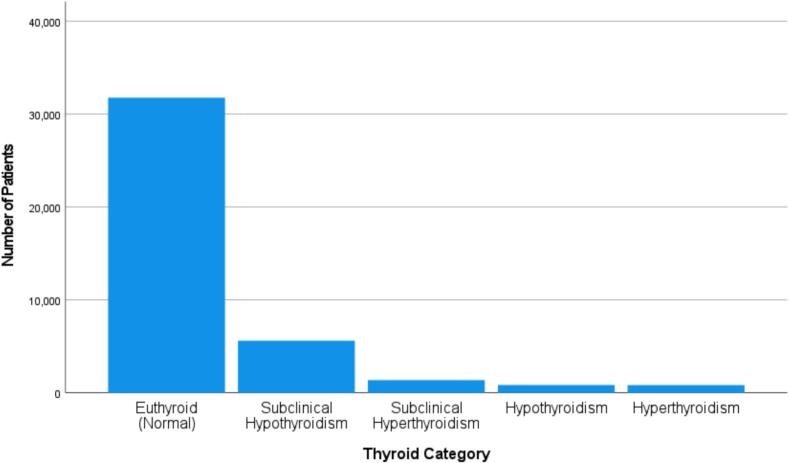
Prevalence of thyroid function categories in the study population(n = 40,390).

Among the total sample, 78.7 % were euthyroid, indicating normal thyroid function. Subclinical hypothyroidism was the most prevalent thyroid disorder, observed in 13.9 % of subjects. This was followed by subclinical hyperthyroidism (3.4 %), overt hyperthyroidism (2.1 %), and overt hypothyroidism (2.1 %) (Fig. 1).

### 3.3 Descriptive statistics of thyroid and lipid markers

The mean serum TSH concentration was 3.4 ± 6.2 μIU/mL. Mean Free T4 and Free T3 concentrations were 16.5 ± 5.1 pmol/L and 6.4 ± 5.8 pmol/L, respectively. The average total cholesterol level was 4.8 ± 1.3 mmol/L, with LDL cholesterol at 2.8 ± 1.1 mmol/L and triglycerides at 1.6 ± 1.1 mmol/L. TPO antibody titers were elevated, with a mean value of 165.4 ± 296.7 IU/mL. Detailed descriptive statistics for lipid parameters and thyroid function tests are presented in Supplementary Table S2.

### 3.4 Distribution of thyroid disorders by age group

**Fig. 2:**
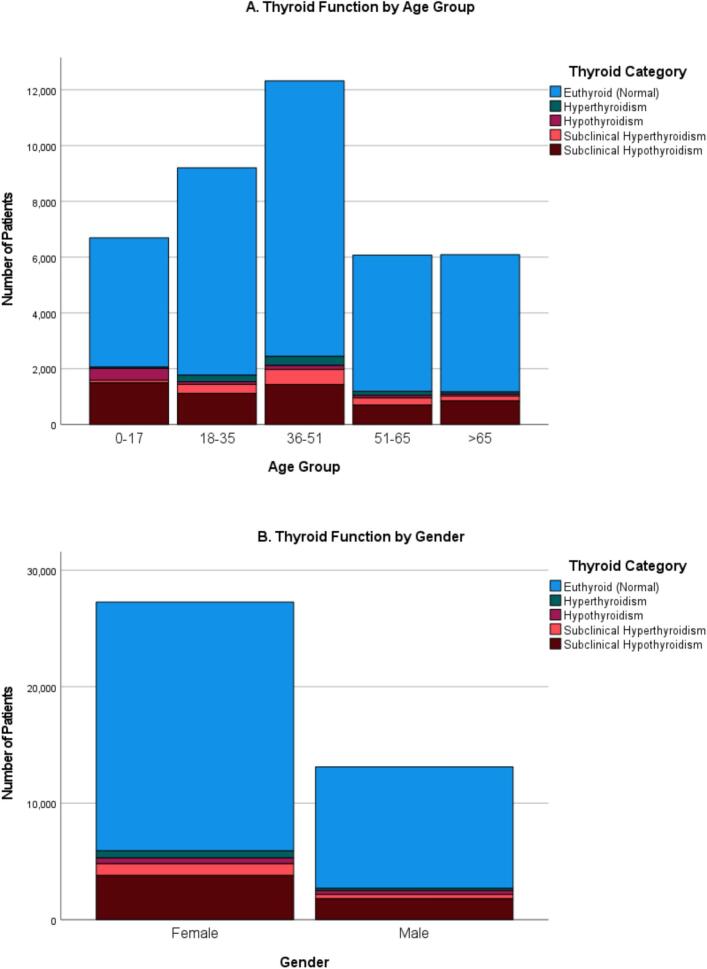
Distribution of thyroid function categories by age and gender. (A) Thyroid function status across five age groups (0–17, 18–35, 36–50, 51–65, >65 years). Subclinical hypothyroidism was most common among adolescents (0–17 years), while hyperthyroidism peaked in the 36–50 years group. (B) Thyroid function status by gender. Thyroid dysfunction was more prevalent in females, particularly subclinical hypothyroidism. Note: Euthyroid = normal thyroid function; Subclinical Hypothyroidism = elevated TSH with normal FT4; Subclinical Hyperthyroidism = suppressed TSH with normal FT4; Overt Hypothyroidism = elevated TSH with low FT4; Overt Hyperthyroidism = suppressed TSH with elevated FT4.

The distribution of thyroid dysfunction varied significantly across age groups (Fig. 2). The 0–17 years group showed the highest prevalence of hypothyroidism and subclinical hypothyroidism. In contrast, hyperthyroidism and subclinical hyperthyroidism were most common in the 36–50 years group. Adults over 65 years were more likely to be euthyroid or have subclinical hypothyroidism. A chi-square analysis confirmed a statistically significant association between age group and thyroid status (χ^2^ = 1519.6, *p* < 0.001).

### 3.5 Thyroid dysfunction and gender

**Fig. 3:**
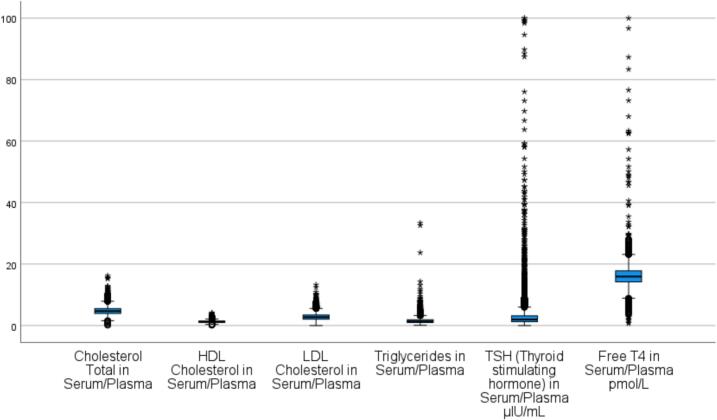
Boxplots of biochemical parameters.

As shown in Fig. 3, thyroid disorders were significantly more common in females than males across all categories. Notably, 74 % of patients with hyperthyroidism and subclinical hyperthyroidism were female. However, males demonstrated a relatively higher proportion of overt hypothyroidism (40.1 %), which may suggest underdiagnosis or lower screening rates among men. The association between gender and thyroid disorder status was statistically significant (χ^2^ = 65.3, p < 0.001).

### 3.6 Correlation between thyroid function and lipid profiles

**Fig. 4:**
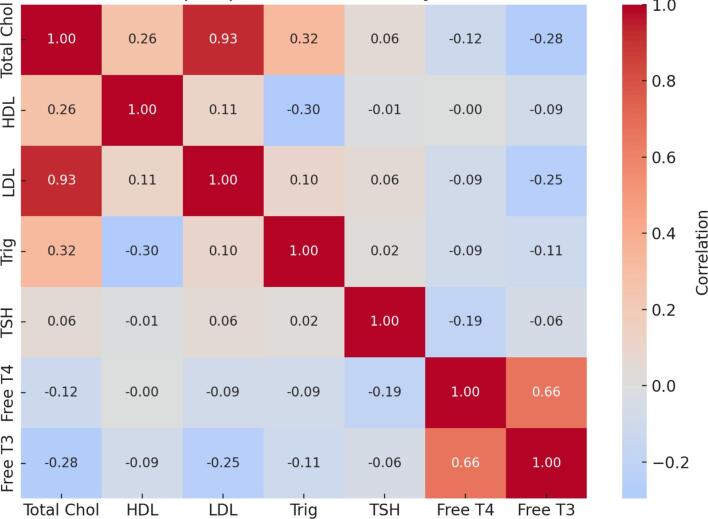
Correlation heatmap of lipid profiles and thyroid function tests (TFTs).

Fig. 4 and Table S4 presents correlations between thyroid hormones and lipid parameters. A strong positive correlation was observed between total cholesterol and LDL cholesterol (*r* = 0.93, *p* < 0.01). Moderate positive correlations were also found between total cholesterol and HDL cholesterol (*r* = 0.26, p < 0.01) and between cholesterol and triglycerides (*r* = 0.32, p < 0.01). An inverse correlation was seen between HDL cholesterol and triglycerides (*r* = −0.30, *p* < 0.01).

TSH showed weak but statistically significant positive associations with total cholesterol and LDL cholesterol (*r* = 0.06 each; p < 0.01). Free T4 was negatively correlated with total cholesterol (*r* = −0.12), LDL cholesterol (*r* = −0.091), and triglycerides (*r* = −0.093), all statistically significant (p < 0.01). Similarly, Free T3 showed negative correlations with total cholesterol (*r* = −0.28) and LDL (*r* = −0.25; *p* < 0.05). A strong positive correlation was observed between Free T3 and Free T4 (*r* = 0.661, p < 0.01), highlighting their interdependence.

### 3.7 Age-related variation in lipid and thyroid parameters

Table 2 summarizes the variation in lipid and thyroid markers across age groups. The 36–50 years age group had the highest total cholesterol levels (5.1 ± 1.2 mmol/L, *p* < 0.001), while HDL cholesterol was highest among individuals aged 18–35 years (1.3 ± 0.4 mmol/L). LDL cholesterol peaked in the 36–50 years group (3.0 ± 1.0 mmol/L, p < 0.001), whereas triglycerides were most elevated in the 51–65 years group (1.7 ± 0.9 mmol/L).

**Table 2:** Comparison of lipid profiles and thyroid function tests by age group (one-way ANOVA).

Parameter	0–17 yrs	18–35 yrs	36–50 yrs	51–65 yrs	>65 yrs	p-value
Total Cholesterol (mmol/L)	4.5 ± 1.4	4.9 ± 1.2	5.1 ± 1.2	4.9 ± 1.3	4.4 ± 1.3	<0.001***
HDL Cholesterol (mmol/L)	1.3 ± 0.4	1.3 ± 0.4	1.3 ± 0.4	1.3 ± 0.4	1.2 ± 0.4	<0.001***
LDL Cholesterol (mmol/L)	2.5 ± 1.2	2.9 ± 1.0	3.0 ± 1.0	2.8 ± 1.1	2.5 ± 1.1	<0.001***
Triglycerides (mmol/L)	1.5 ± 2.2	1.4 ± 1.2	1.7 ± 1.3	1.7 ± 0.9	1.5 ± 0.8	<0.001***
TSH (μIU/mL)	4.9 ± 7.7	3.1 ± 5.8	3.0 ± 5.8	3.0 ± 6.1	3.1 ± 5.6	<0.001***
Free T4 (pmol/L)	17.5 ± 3.8	16.6 ± 5.8	16.2 ± 5.4	16.2 ± 5.0	16.3 ± 4.4	<0.001***
Free T3 (pmol/L)†	7.8 ± 9.5	7.0 ± 6.1	5.9 ± 5.2	6.4 ± 4.5	4.2 ± 1.2	0.283
TPO Antibodies (IU/mL)†	0.8 ± 0.4	0.8 ± 0.4	0.9 ± 0.3	0.9 ± 0.3	0.8 ± 0.4	0.006*

Note: Data presented as mean ± SD. †Values available for a limited subset of patients. ***p < 0.001, *p < 0.05.

TSH levels were highest in the 0–17 years group (4.9 ± 7.7 μIU/mL, *p* < 0.001), while Free T4 concentrations also peaked in this group (17.5 ± 3.8 pmol/L). No significant differences in Free T3 levels were found across age groups (*p* = 0.283). TPO antibody levels were highest in the 36–50 years group (0.9 ± 0.3 IU/mL, *p* = 0.006), suggesting increased autoimmune activity within this demographic.

### 3.8 Gender-based differences in lipid and thyroid parameters

Table 3 highlights gender-related differences in biochemical markers. Female participants had higher levels of total cholesterol (4.9 ± 1.2 mmol/L vs. 4.7 ± 1.3 mmol/L, *p* < 0.001), HDL cholesterol (1.4 ± 0.4 vs. 1.2 ± 0.3 mmol/L, p < 0.001), and LDL cholesterol (2.8 ± 1.1 vs. 2.8 ± 1.2 mmol/L, *p* = 0.002) compared to males. In contrast, triglyceride levels were significantly higher in males (1.7 ± 1.3 mmol/L vs. 1.5 ± 1.0 mmol/L, p < 0.001), indicating a trend toward male-predominant hypertriglyceridemia.

**Table 3:** Comparison of lipid profiles and thyroid function tests by gender (independent samples *t*-test).

Parameter	Female (mean ± SD)	Male (mean ± SD)	p-value
Total Cholesterol (mmol/L)	4.9 ± 1.2	4.7 ± 1.3	<0.001***
HDL Cholesterol (mmol/L)	1.4 ± 0.4	1.2 ± 0.3	<0.001***
LDL Cholesterol (mmol/L)	2.8 ± 1.1	2.8 ± 1.2	0.002**
Triglycerides (mmol/L)	1.5 ± 1.0	1.7 ± 1.3	<0.001***
TSH (μIU/mL)	3.3 ± 6.1	3.4 ± 6.5	0.095
Free T4 (pmol/L)	16.3 ± 5.2	16.9 ± 4.8	<0.001***
Free T3 (pmol/L)†	6.1 ± 4.9	7.3 ± 7.4	0.080
TPO Antibodies (IU/mL)†	188.1 ± 307.1	120.8 ± 270.5	0.026*

Note: Data presented as mean ± SD. †Values available for a limited subset of patients. ***p < 0.001, **p < 0.01, *p < 0.05.

Free T4 levels were significantly higher in males (16.9 ± 4.8 pmol/L vs. 16.3 ± 5.2 pmol/L, p < 0.001), while no statistically significant difference was observed in Free T3 levels between genders (*p* = 0.080). TSH levels showed no significant gender difference (*p* = 0.095). However, TPO antibodies were substantially elevated in females (188.1 ± 307.1 IU/mL vs. 120.8 ± 270.5 IU/mL; *p* = 0.026), supporting a greater prevalence of autoimmune thyroid activity in women.

## 4 Discussion

This large retrospective study from a tertiary care hospital in Al Batinah North, Oman, found that 21.3 % of patients undergoing thyroid testing had some form of thyroid dysfunction, most commonly subclinical hypothyroidism. Thyroid dysfunction was more frequent in females and showed distinct age patterns. Statistically significant but weak associations were observed between thyroid hormones and lipid parameters. While these findings add to existing Omani and regional data, they must be interpreted cautiously given the study's hospital-based, retrospective design.

The observed prevalence was slightly higher than the pooled Middle Eastern estimates reported by Kargar et al. [2]. However, this difference is likely explained by selection and referral bias, as patients in tertiary centres are more likely to undergo thyroid testing. Consequently, the results cannot be generalized to the wider Omani population. The higher prevalence observed in this study may also reflect regional trends, including the rising incidence of non-communicable diseases such as diabetes, obesity, and chronic kidney disease (CKD), all of which have been linked to thyroid dysfunction. Al-Fahdi et al. [8] reported thyroid abnormalities in 11.7 % of Omani patients with CKD, with subclinical hypothyroidism accounting for nearly two-thirds of cases. Similarly, Al-Sumry et al. [20] found thyroid dysfunction in 12.6 % of Omani diabetic patients, particularly in those with poor glycaemic control. Data from Pakistan by Hussain Awan et al. [6] also confirm high prevalence among diabetics. Collectively, these findings support the established association between thyroid dysfunction and chronic metabolic conditions, emphasizing the importance of considering thyroid testing in chronic disease management.

One notable finding in this study was the high prevalence of subclinical hypothyroidism among adolescent and reproductive-age females, accounting for 67.5 % of cases. This observation is consistent with regional and international studies, which report a higher incidence of thyroid dysfunction among women [20,22]. It also raises questions about the applicability of international guidelines such as those of the U.S. Preventive Services Task Force and the American Thyroid Association, which do not recommend routine screening in asymptomatic individuals. Given potential differences in iodine intake, autoimmune predisposition, and healthcare-seeking behavior in the Gulf region, further evaluation is needed to determine whether context-specific screening approaches are appropriate. In our cohort, elevated TPO antibody titres among females further support an autoimmune etiology, consistent with Hashimoto's thyroiditis [9]. Future research is needed to evaluate whether tailored screening approaches for women in the Gulf region would be justified.

Age-stratified analysis showed that hypothyroidism and subclinical hypothyroidism were more common in individuals aged 0–17 years, whereas hyperthyroidism predominated in the 36–50-year age group. This pattern contrasts with published reports where thyroid dysfunction is typically more prevalent among middle-aged or elderly populations. For example, Al-Sumry et al. [17] observed higher rates of subclinical hypothyroidism in Omani adults over 40 years. Our finding of increased hypothyroidism among adolescents should therefore be interpreted cautiously, as it may reflect referral or testing bias in this age group. Nonetheless, given the critical role of thyroid hormones in growth and neurodevelopment, this observation warrants further investigation through population-based pediatric studies [21].

The observed associations between thyroid dysfunction and lipid parameters were consistent with existing evidence on the role of thyroid hormones in lipid metabolism. Free T4 demonstrated a negative correlation with total cholesterol, LDL cholesterol, and triglycerides, suggesting that reduced thyroid hormone activity may contribute to dyslipidaemia even in subclinical states. These findings agree with those of Atrooz et al. [7], who reported abnormal lipid profiles among hypothyroid patients in Jordan, although their correlations were not significant across all categories. In our study, correlations between TSH and lipid levels were weaker but statistically significant, indicating a possible link, though effect sizes were small and of limited clinical significance.

Gender-based differences were also observed in both thyroid and lipid parameters. Females had higher levels of total cholesterol, HDL, and LDL, while males had significantly higher triglycerides and free T4. Similar trends have been described by Al-Sumry et al. [20] and Duntas and Brenta [13]. These differences may reflect hormonal influences, fat distribution patterns, and gender-specific metabolic regulation. Although exploratory, such findings may have implications for refining risk assessment in future research, including potential consideration of gender-specific thresholds.

Although comorbidities such as diabetes and CKD were not directly analyzed in this cohort, existing literature strongly supports their association with thyroid dysfunction [17]. In CKD, overlapping symptoms such as fatigue, weight changes, and cold intolerance may obscure thyroid-related abnormalities, complicating diagnosis. These overlaps underscore the importance of considering thyroid function testing in multidisciplinary care for patients with chronic disease.

### 4.1 Implications for clinical practice and public health

The findings of this hospital-based study suggest several potential implications for endocrinology and public health in Oman and similar contexts. The relatively high prevalence of subclinical hypothyroidism, particularly among females and adolescents, points to a metabolically vulnerable but often clinically silent subgroup. While routine population-wide screening cannot be recommended based on these data, targeted testing in high-risk groups such as women of reproductive age, adolescents with growth or developmental concerns, and patients with chronic disease may warrant further exploration in future community-based studies.

The observed associations between thyroid hormone levels (especially free thyroxine and free triiodothyronine) and lipid parameters suggest that thyroid dysfunction could serve as an early marker of metabolic risk [17], but these required validation in prospective studies. In clinical practice, integrating thyroid testing with lipid profiling may help identify at-risk patients earlier, although this requires validation in prospective studies before being adopted into practice guidelines.

At the public health level, awareness campaigns and education programs could improve recognition of thyroid-related symptoms, encourage timely healthcare seeking, and highlight the potential overlap between thyroid disease and common chronic conditions such as obesity, diabetes, and cardiovascular disease. Importantly, the apparent high prevalence of hypothyroidism among adolescents in this cohort should be interpreted cautiously, as it may reflect referral and testing patterns rather than a true epidemiological trend. Dedicated pediatric, population-based studies are needed to confirm this observation.

### 4.2 Strengths and limitations

A major strength of this study is its large sample size and standardized laboratory methods. However, several important limitations restrict interpretability. First, the retrospective design precludes causal inference. Second, the hospital-based sample introduces referral bias, with overrepresentation of women and adolescents who may have greater access to testing. Third, missing data on comorbidities, medications, and thyroid antibodies limit adjustment for confounding. Fourth, iodine status was not assessed, despite its relevance in Oman, where regional variation in iodine sufficiency persists. Finally, correlations between thyroid function and lipid levels were statistically significant but small in magnitude, limiting clinical relevance. Reverse causation cannot be excluded, and the absence of multivariable regression further reduces the strength of inference.

### 4.3 Future research directions

Future studies should adopt prospective, multicentre designs across diverse regions of Oman and the wider Gulf to validate and extend the present findings. Such research should include detailed assessments of dietary intake, iodine status, comorbidities, physical activity, and medication use, enabling clearer characterization of causal relationships and risk factors for thyroid dysfunction. Further investigations are warranted on the reproductive consequences of thyroid dysfunction, particularly in adolescent girls and women of childbearing age, as well as the neurocognitive and psychosocial impacts of undiagnosed hypothyroidism in younger populations. Longitudinal pediatric studies are especially needed to clarify these outcomes.

At the biological level, genetic and immunological profiling such as HLA typing, TSH receptor antibody testing, and TPO antibody subtyping could help identify high-risk subgroups and refine etiological classification. Cardiovascular outcomes also require further evaluation, particularly the long-term risks associated with subclinical thyroid dysfunction. Randomised controlled trials assessing early treatment strategies in such patients would help guide evidence-based management protocols.

From a health systems perspective, economic analyses are needed to evaluate the cost-effectiveness of integrating thyroid function testing with lipid profiling in primary care. Future research should also align with broader efforts to strengthen evidence-based decision-making in the region, as highlighted in recent reviews of continuing medical education and knowledge translation.

## 5 Conclusion

In this large hospital-based cohort from Al Batinah North, Oman, thyroid dysfunction was common, with subclinical hypothyroidism being the most frequent abnormality. Age- and gender-specific patterns were observed, and weak but statistically significant associations with lipid parameters were identified. These findings provide insight into thyroid disease trends in a clinical population but cannot be generalized to the wider Omani population. While the results raise important questions about at-risk subgroups such as women and adolescents, further population-based studies are needed before targeted screening or management strategies can be recommended.

## CRediT authorship contribution statement

**Vijaya Marakala:** Writing – review & editing, Writing – original draft, Visualization, Validation, Supervision, Project administration, Methodology, Investigation, Formal analysis, Data curation, Conceptualization. **Gulam Saidunnisa Begum:** Writing – review & editing, Validation, Methodology, Data curation. **Salima Al Maqbali:** Writing – review & editing, Visualization, Methodology, Formal analysis, Data curation. **Elham Said Ahmed Al Risi:** Writing – review & editing, Software, Methodology, Investigation, Formal analysis, Data curation.

## Declaration

Data are available upon reasonable request, subject to ethical approval from the Institutional Ethics Committee.

This research did not receive any specific grant from funding agencies in the public, commercial, or not-for-profit sectors.

## Declaration of competing interest

The authors declare that they have no known competing financial interests or personal relationships that could have appeared to influence the work reported in this paper.

## Appendix A Supplementary data

Supplementary material Image 1
