# Supplementary material for: Prevalence of thyroid disorders in a tertiary care hospital in Al Batinah North Governorate, Oman
Source: Dialogues Health. 2025 Oct 2;7:100246. doi: 10.1016/j.dialog.2025.100246 (PMC12538129; doi:10.1016/j.dialog.2025.100246)
Supplement: Supplementary file 1 — Supplementary material [file mmc1.docx]

**Supplementary Tables**

Supplementary Table S1. Nationality distribution of patients undergoing thyroid function testing (n = 40,390).

|  | Nationality | Number | Percentage |
| --- | --- | --- | --- |
| **Nationality** | AMERICAN | 3 | .0 |
|  | BAHRAINI | 10 | .0 |
|  | BANGLADESHI | 123 | .3 |
|  | BENIN | 1 | .0 |
|  | CANADIAN | 4 | .0 |
|  | EGYPTIAN | 347 | .9 |
|  | EMIRATES | 28 | .1 |
|  | ETHOPIAN | 4 | .0 |
|  | FILIPINO | 43 | .1 |
|  | GERMAN | 1 | .0 |
|  | GHANIAN | 3 | .0 |
|  | INDIAN | 910 | 2.3 |
|  | INDONESIAN | 4 | .0 |
|  | IRANI | 29 | .1 |
|  | IRAQI | 33 | .1 |
|  | JORDANIAN | 9 | .0 |
|  | KENYAN | 1 | .0 |
|  | KUWAITI | 2 | .0 |
|  | LEBANEES | 2 | .0 |
|  | LIBYAN | 1 | .0 |
|  | MOROCCAN | 26 | .1 |
|  | NEPALEES | 4 | .0 |
|  | NIGERIAN | 2 | .0 |
|  | OMANI | 38366 | 95.0 |
|  | PAKISTANI | 178 | .4 |
|  | PALESTINIAN | 11 | .0 |
|  | POLISH | 2 | .0 |
|  | PORTUGEES | 1 | .0 |
|  | SAUDI | 7 | .0 |
|  | SOUTH AFRICAN | 1 | .0 |
|  | SRI LANKAN | 13 | .0 |
|  | SUDANEES | 106 | .3 |
|  | SYRIAN | 36 | .1 |
|  | TAIWANEES | 1 | .0 |
|  | TANZANIAN | 5 | .0 |
|  | TUNISIAN | 31 | .1 |
|  | TURKISH | 1 | .0 |
|  | UGANDAN | 5 | .0 |
|  | UKRAINE | 1 | .0 |
|  | VENEZUELAN | 1 | .0 |
|  | YEMENI | 33 | .1 |
|  | ZAMBIAN | 1 | .0 |
|  | **Total** | **40390** | **100.0** |

**Supplementary Table S2. Descriptive statistics of biochemical parameters.**

| Variable | N | Mean | SD | Min | Max |
| --- | --- | --- | --- | --- | --- |
| Total Cholesterol (mmol/L) | 12,311 | 4.8 | 1.3 | 0.1 | 19.6 |
| HDL Cholesterol (mmol/L) | 12,299 | 1.3 | 0.4 | 0.1 | 4.2 |
| LDL Cholesterol (mmol/L) | 12,165 | 2.8 | 1.1 | 0.0 | 13.3 |
| Triglycerides (mmol/L) | 12,217 | 1.6 | 1.1 | 0.1 | 42.0 |
| TSH (µIU/mL) | 39,395 | 3.4 | 6.2 | 0.01 | 153.2 |
| Free T4 (pmol/L) | 39,767 | 16.5 | 5.1 | 0.5 | 100.0 |
| Free T3 (pmol/L) * | 298 | 6.4 | 5.8 | 0.8 | 47.5 |
| TPO Antibodies (IU/mL) * | 429 | 165.4 | 296.7 | 1.0 | 1300 |

Mean, standard deviation (SD), minimum, and maximum values are presented for serum lipid parameters and thyroid function tests (TFTs). Data availability varied across parameters, with fewer results for free T3 and thyroid peroxidase (TPO) antibodies compared to TSH and free T4.

**Supplementary Table S3. Distribution of thyroid function by gender (n = 40,390).**

| Gender | Euthyroid n (%) | Subclinical Hypothyroidism n (%) | Subclinical Hyperthyroidism n (%) | Overt Hypothyroidism n (%) | Overt Hyperthyroidism n (%) | Total n (%) |
| --- | --- | --- | --- | --- | --- | --- |
| Female | 21,357 (78.3) | 3,791 (13.9) | 1,011 (3.7) | 501 (1.8) | 607 (2.2) | 27,267 (67.5) |
| Male | 10,411 (79.3) | 1,809 (13.8) | 355 (2.7) | 335 (2.6) | 213 (1.6) | 13,123 (32.5) |
| Total | 31,768 (78.7) | 5,600 (13.9) | 1,366 (3.4) | 836 (2.1) | 820 (2.0) | 40,390 (100) |

Note: Chi-square = 65.3, p < 0.001 (association between gender and thyroid function categories). Gender-specific frequencies and percentages of thyroid function categories. Thyroid dysfunction was more prevalent in females compared to males across most categories.

**Supplementary Table S4. Correlation between lipid profiles and thyroid function tests (TFTs).**

| Variable | Total Cholesterol | HDL | LDL | Triglycerides | TSH | Free T4 | Free T3† |
| --- | --- | --- | --- | --- | --- | --- | --- |
| Total Cholesterol | 1.00 | .262** | .925** | .317** | .056** | –.118** | –.275* |
| HDL | – | 1.00 | .110** | –.296** | –.012 | –.003 | –.091 |
| LDL | – | – | 1.00 | .101** | .056** | –.091** | –.253* |
| Triglycerides | – | – | – | 1.00 | .018* | –.093** | –.112 |
| TSH | – | – | – | – | 1.00 | –.193** | –.056 |
| Free T4 | – | – | – | – | – | 1.00 | .661** |
| Free T3† | – | – | – | – | – | – | 1.00 |

Note: Pearson correlation coefficients are shown.
†Free T3 data were available for a limited subset (n = 298).
**p < 0.01, *p < 0.05. Pearson correlation coefficients (r) between serum lipid parameters (total cholesterol, HDL, LDL, triglycerides) and thyroid function tests (TSH, free T4, free T3). Most correlations were statistically significant but weak in magnitude. Free T3 values were available only for a subset of patients.
